# ΔNp63α Transcriptionally Regulates the Expression of CTEN That Is Associated with Prostate Cell Adhesion

**DOI:** 10.1371/journal.pone.0147542

**Published:** 2016-01-19

**Authors:** Kuan Yang, Wei-Ming Wu, Ya-Chi Chen, Su Hao Lo, Yi-Chun Liao

**Affiliations:** 1 Department of Biochemical Science and Technology, National Taiwan University, Taipei, Taiwan; 2 Department of Biochemistry and Molecular Medicine, University of California-Davis, Sacramento, California, United States of America; Università degli Studi di Milano, ITALY

## Abstract

p63 is a member of the p53 transcription factor family and a linchpin of epithelial development and homeostasis. p63 drives the expression of many target genes involved in cell survival, adhesion, migration and cancer. In this study, we identify C-terminal tensin-like (CTEN) molecule as a downstream target of ΔNp63α, the predominant p63 isoform expressed in epithelium. CTEN belongs to the tensin family and is mainly localized to focal adhesions, which mediate many biological events such as cell adhesion, migration, proliferation and gene expression. Our study demonstrate that ΔNp63 and CTEN are both highly expressed in normal prostate epithelial cells and are down-regulated in prostate cancer. In addition, reduced expression of *CTEN* and *ΔNp63* is correlated with prostate cancer progression from primary tumors to metastatic lesions. Silencing of ΔNp63 leads to decreased mRNA and protein levels of CTEN. ΔNp63α induces transcriptional activity of the *CTEN* promoter and a 140-bp fragment upstream of the transcription initiation site is the minimal promoter region required for activation. A putative binding site for p63 is located between -61 and -36 within the *CTEN* promoter and mutations of the critical nucleotides in this region abolish ΔNp63α-induced promoter activity. The direct interaction of ΔNp63α with the *CTEN* promoter was demonstrated using a chromatin immunoprecipitation (ChIP) assay. Moreover, impaired cell adhesion caused by ΔNp63α depletion is rescued by over-expression of CTEN, suggesting that CTEN is a downstream effector of ΔNp63α-mediated cell adhesion. In summary, our findings demonstrate that ΔNp63α functions as a trans-activation factor of *CTEN* promoter and regulates cell adhesion through modulating CTEN. Our study further contributes to the potential regulatory mechanisms of CTEN in prostate cancer progression.

## Introduction

p63 belongs to the p53 transcription factor family, which also includes p73, and it has a structure similar to that of p53 [1–4]. The p63 protein contains N-terminal transactivation (TA), DNA-binding and oligomerization domains [3]. Due to differential promoter usage, the *p63* gene generates transcripts encoding two isoforms, TAp63 with a p53-like TA domain and ΔNp63 with a truncated N-terminus [3,5]. In addition, alternative splicing at the 3’ end of the primary RNA transcripts of TAp63 and ΔNp63 produce α, β, γ, δ, and ε splice variants of each isotype [3,6]. However, the six variants with N-terminal TA or ΔN and the C-terminal α, β, γ are the most studied isoforms. TAp63 isoforms are capable of transactivating p53 target genes, whereas ΔNp63 isoforms function as dominant-negative inhibitors of p53 as well as the TAp63 and TAp73 isoforms due to the lack of TA domain [3]. However, ΔNp63 variants are not transcriptionally inactive and contain a unique N-terminal amino acid sequence that contributes to their transcriptional activity, allowing them to act as transcriptional activators or repressors [7–11].

While *p53* is a well-known tumor suppressor gene and frequently inactively mutated or deleted in human cancers, *p63* plays a key role in regulating epithelial development and homeostasis [12–15]. The p63 protein is highly expressed in a variety of epithelial tissues [3,16] and p63-knockout mice display profound developmental defects in limbs, skin and other stratified epithelia. Knockout mice also lack epithelial appendages, such as mammary glands, salivary glands, hair follicles and teeth [13,14]. In humans, heterozygous germline mutations of p63 cause less severe but similar ectodermal dysplastic syndromes [17–19]. The expression of p63 in mice is mainly detected within the primitive ectoderm prior to stratification and continues to be expressed through embryogenesis [14,20–22]. Detailed studies on the expression levels of p63 isoforms have indicated that ΔNp63 is required for the maturation of embryonic epidermis and the maintenance of the basal layer, whereas TAp63 is essential for the initiation of epithelial stratification [23–26].

ΔNp63α is the predominant isoform in the basal compartment of stratified epithelia [16,25,27–29]. ΔNp63α plays a critical role in the regulation of epithelial cell adhesion and depletion of ΔNp63α expression induces cell detachment and anoikis [30–33]. However, the role of ΔNp63α in tumorigenesis is complex. ΔNp63α is commonly overexpressed in squamous epithelial cancers but lost in other tumor types, such as bladder cancer and adenocarcinoma of the breast and prostate [16,34–38]. Some studies have implicated that ΔNp63α is oncogenic [39–41] while others have indicated that ΔNp63α regulates key targets involved in tumor suppression [42–48]. Therefore, identification of ΔNp63α target genes is essential for shedding light on the functions of ΔNp63α in epithelial integrity and tumor progression.

The *CTEN* gene is a likely ΔNp63α target in keratinocytes, but the regulatory mechanism remains largely unknown [49]. CTEN (C-terminal tensin-like protein, aka tensin4, TNS4) is the smallest protein in the tensin family and is mainly localized to focal adhesions. CTEN contains the SH2-PTB domains similar to other tensins but lacks the conserved N-terminal actin-binding domain [50]. Although studies of CTEN mutant or knockout mice have not been reported, several lines of evidence have demonstrated that CTEN plays an important role in the regulation of cell motility, apoptosis, tumorigenicity, growth factor receptor homeostasis and renal tubulogenesis [51]. However, these regulatory mechanisms are likely to be tissue context dependent. In normal tissues, the expression pattern of CTEN is distinctive. *CTEN* mRNA levels are highly enriched in prostate and placenta but barely detectable in other tissues [50]. A functional promoter region of human *CTEN* gene has been identified and a 327-bp fragment around exon1 confers significantly strong transcriptional activity in prostate epithelial cell lines [52]. Moreover, CTEN is highly expressed in prostate epithelial cells and significantly down-regulated in prostate cancer [50,52,53]. CTEN protein expression is inversely correlated with pathological Gleason scores of prostate cancer patients and decreased CTEN is associated with resistance to chemotherapeutic agents [53]. These findings suggest that CTEN expression levels might govern the transformation of normal prostate cells into cancer cells. Therefore, investigation of the mechanism regulating CTEN expression will offer a better understanding of the molecular events involved in the process of cell transformation.

In the present study, we have investigated the association between ΔNp63α and CTEN expression as well as their functional relationship in the prostate. Our analyses of their expression levels in human prostate cancer datasets, cell lines and tissue samples revealed a novel correlation between ΔNp63α and CTEN. We also demonstrated that ΔNp63α activates the transcription of *CTEN* through direct binding to the *CTEN* promoter. Moreover, we found that CTEN is a downstream effector of ΔNp63-mediated cell adhesion. Our work highlights a novel ΔNp63α target, CTEN, which is important for the regulation of prostate cell adhesion and is associated with prostate cancer progression.

## Materials and Methods

### Bioinformatics analyses

The microarray data set of Grasso prostate was acquired from the Oncomine online database (http://www.oncomine.org) by selecting the filter of prostate cancer and the *TP63* gene under coexpression analysis. Pearson's correlation was used as a measure of correlation between *TP63* and *TNS4* (*CTEN*) gene profiles in the Grasso prostate data set. The GSE3325 gene expression data set was downloaded from Gene Expression Omnibus (GEO, http://www.ncbi.nlm.nih.gov/geo/). The gene expression profiles were presented in the form of box-and-whisker plots. The relative expression ranking was used to represent mRNA levels.

### Cell culture

RWPE-1 cells (American Type Culture Collection, ATCC, Manassas, VA, USA) were cultured in keratinocyte serum free media (Invitrogen, Carlsbad, CA, USA) supplemented with 0.05 mg/mL bovine pituitary extract, 5 ng/mL human recombinant epidermal growth factor and 1% penicillin/streptomycin. 22Rv1 and LNCaP cells purchased from Bioresource Collection and Research Center (BCRC, Hsinchu, Taiwan) were cultured in RPMI1640 media (Invitrogen) supplemented with 10% fetal bovine serum (FBS) and 1% penicillin/streptomycin. PC-3 cells (BCRC) were cultured in F-12 media (Invitrogen) supplemented with 10% FBS and 1% penicillin/streptomycin. DU-145 (BCRC) and HEK293 (ATCC) cells were cultured in DMEM/high glucose media (Invitrogen) supplemented with 10% FBS and 1% penicillin/streptomycin. All cells were maintained at 37°C in a humidified atmosphere containing 5% CO_2_.

### RNA extraction and quantitative real-time polymerase chain reaction (qPCR)

Total RNA was isolated using the RNAspin Mini Kit (GE Healthcare, Pittsburgh, PA, USA) according to the instructions in the user manual. cDNA was synthesized from 1 μg RNA using the Transcription First Strand cDNA Synthesis Kit (Roche, Indianapolis, IN, USA) according to the manufacturer’s instructions. qPCR analyses were performed in a CFX Connect System using the iQ SYBR Green Supermix (Bio-Rad, Hercules, CA, USA). The primers used for qPCR are listed in S1 Table. Relative quantification of gene expression was normalized against 18S rRNA using the ΔC_T_ method.

### Protein preparation and immunoblotting

Cells were collected and suspended in cell lysis buffer (50 mM Tris/pH 7.5, 150 mM NaCl, 5 mM EDTA, 1% TritonX-100, and a protease inhibitor cocktail). After centrifugation at 13000×g, 4°C for 15 minutes, the supernatant was collected as the total protein lysate. Samples were subjected to SDS-PAGE and transferred to PVDF membranes followed by blocking using Gelatin-NET (0.25% w/v gelatin, 50 mM Tris/pH 8.0, 150 mM NaCl, 5 mM EDTA, and 0.05% v/v Tween-20). Immunoblotting was then performed using Gelatin-NET-diluted primary antibodies against p40 (which recognizes all ΔNp63 isoforms except the TAp63 isoforms; Oncogene, Boston, MA, USA), CTEN (Spring Bioscience, Pleasanton, CA, USA), or α-tubulin (Sigma-Aldrich, St. Louis, MO, USA) followed by incubation with the appropriate peroxidase-linked secondary antibody (KPL, Gaithersburg, MD, USA). Protein signals were detected by Chemiluminescent HRP Substrate (Millipore, Billerica, MA, USA) and visualized using the BioSpectrum Imaging System (UVP, Upland, CA, USA).

### siRNA mediated RNA interference

Small interfering RNA (siRNA) against ΔNp63 was obtained from Dharmacon ON-TARGET custom siRNA synthesis (Dharmacon, Lafayette, CO, USA) (sense 5’-ACAAUGCCCAGACUCAAUU-3’-dTdT and antisense 5’-AAUUGAGUCUGGGCAUUGU-3’-dTdT). CTEN-specific siRNA was purchased from Invitrogen. MISSION siRNA Universal Negative Control (Sigma-Aldrich) was used as a control in all the knockdown experiments. siRNAs were transfected into RWPE-1 cells by Lipofectamine 2000 (Invitrogen) according to manufacturer’s instructions. Cells were harvested for further analysis 48 h after transfection.

### Plasmid constructs and luciferase assay

ΔNp63β (#27041) and FLAG-tagged TAp63α (#27008), ΔNp63α (#26979), ΔNp63γ (#27012) plasmids were purchased from Addgene (Cambridge, MA, USA). Construction of the FLAG-tagged CTEN and pGL-290 plasmids were described previously [52,54]. Different lengths of 5’ flanking DNA sequence of the *CTEN* gene were amplified by PCR using genomic DNA isolated from RWPE-1 cells. The PCR products were subcloned into the pSCA vector (Clontech, Mountain View, CA, USA) by TA cloning. After sequence verification, the fragments were directionally subcloned into *Xho*I and *Hin*dIII sites in a promoterless, enhancerless pGL3-Basic luciferase reporter vector (Promega, Madison, WI, USA). These constructs are designated as pGL-923, pGL-539 and pGL-483 hereafter. pGL-140 (or pGL-140-wt) was generated by ligation of the 5-kb fragment isolated from *Sac*I-digested pGL-290. The site-specific mutations on pGL-140-m were introduced into the pGL-140-wt by site directed mutagenesis.

The pGL3-Basic luciferase reporter vector containing various lengths of the *CTEN* promoter region was co-transfected with the p63 isoform expression plasmid (TAp63α-FLAG, ΔNp63α-FLAG, ΔNp63β or ΔNp63γ-FLAG) or an empty vector into HEK293 cells using TurboFect (Thermo Fisher, Pittsburgh, PA, USA) according to the manufacturer’s instructions. The pRL-TK plasmid (Promega), which contains the *Renilla* luciferase reporter gene, was also cotransfected as an internal control to monitor transfection efficiency. Cells were harvested 48 h after transfection and the luciferase activity was measured using the Dual-Luciferase Reporter Assay System (Promega) according to the manufacturer’s instructions. The individual relative light units (RLU) from the firefly luciferase were normalized to that from the *Renilla* luciferase.

### Chromatin immunoprecipitation (ChIP)

RWPE-1 cells (5×10^6^) were collected and crosslinked by 1% formaldehyde in fresh culture medium at room temperature under shaking for 10 min followed by quenching with 0.125 M glycine. Cells were lysed in ChIP lysis buffer (50 mM Tris/pH 8.0, 10 mM EDTA, 1% SDS, and a protease inhibitor cocktail) and the chromatin was sheared by sonication under conditions yielding fragments ranging from 200 to 1000 bp. The sonicated cell lysate was centrifuged and the supernatant diluted 10-fold using ChIP dilution buffer (16.7 mM Tris/pH 8.0, 150 mM NaCl, 0.01% SDS, 1% TritonX-100, 1.2 mM EDTA, and a protease inhibitor cocktail). After pre-cleaning by Protein A Sepharose (GE Healthcare) and centrifugation, the supernatant was then incubated with 2 μL anti-ΔNp63 antibodies (Oncogene) or 1 μg rabbit IgG (Sigma-Aldrich) overnight at 4°C. Immunoprecipitation was performed at 4°C for 1 hour to isolate antibody-bound chromatin by incubating the supernatant with Protein A Sepharose, previously saturated with sheared salmon sperm DNA (Sigma-Aldrich) and BSA (Bionovas, Bremerton WA, USA). The immunoprecipitated complexes were collected by centrifugation and washed four times with TSE-500 (20 mM Tris/pH 8.0, 500 mM NaCl, 0.1% SDS, 1% Triton X-100, and 2 mM EDTA), twice with LiCl detergent (10 mM Tris/pH 8.0, 1% NP-40, 0.25 M LiCl, 1% sodium deoxycholate, and 1 mM EDTA), and twice with TE buffer (10 mM Tris/pH 8.0 and 1 mM EDTA). ChIP elution buffer (1% SDS and 100 mM NaHCO_3_) was then added to the pellet with rotation for 15 min. The complex crosslinking was reversed by heating the supernatant with 0.2 M NaCl at 65°C overnight. DNA was released by incubation with proteinase K (Sigma-Aldrich) and extracted using the PCR DNA isolation system (Viogene, New Taipei City, Taiwan) according to the manufacturer’s directions.

Purified DNA was subsequently used for conventional PCR and qPCR analyses using specific primers listed in S1 Table. ChIP-qPCR values were first normalized by the respective input values and then fold enrichments were calculated compared with enrichment of a CTEN promoter region not expected to interact with ΔNp63α (-2420 ~ -2300).

### Adhesion assay

RWPE-1 cells were transfected by siRNA (100 pmol) and plasmids (1 μg). Forty-eight hours after transfection, cells were reseeded on a 96-well plate (2×10^4^ cells/well) coated with fibronectin (20 μg/mL, Millipore) and blocked with 5% BSA (Bionovas). Cells were then incubated in 37°C for 1.5 hr. The non-adherent cells were removed by PBS, and the adherent cells were fixed in formaldehyde and stained with crystal violet. The stained cells were incubated in 0.5% Triton X-100 for 10 min for dye extraction and quantified using an ELISA plate reader at 595 nm.

## Results

### Positive correlation between p63 and CTEN expression in human prostate tissues

**Fig 1:**
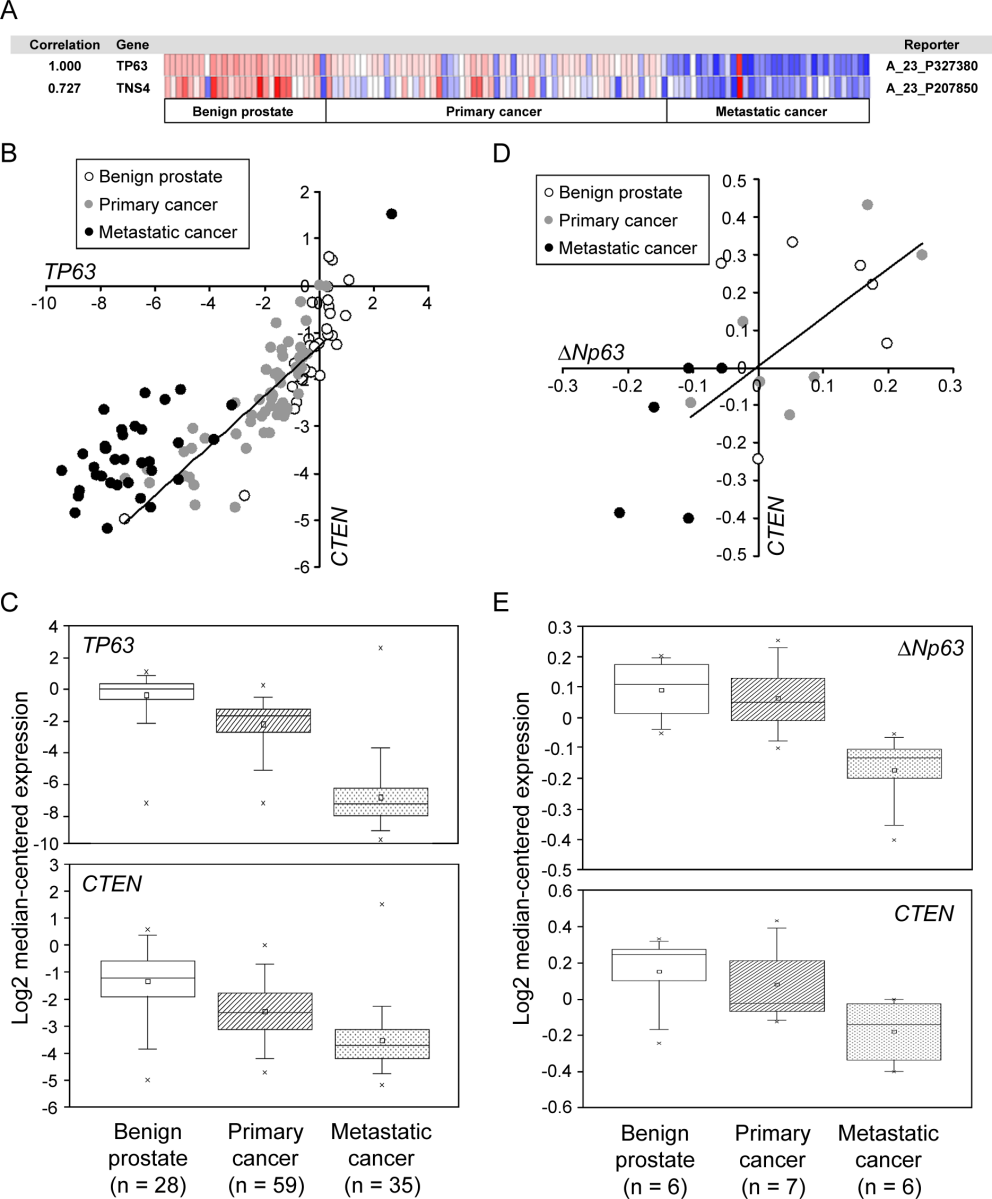
Positive correlation between p63 and CTEN expression in human prostate tissues. (A) The Grasso prostate dataset was analyzed. We used the following filters: gene “TP63”, analysis type, and “coexpression analysis”. Color changes according to different expression levels from the least (blue) to the most (red) are shown with fluctuating color intensity. *CTEN* (*TNS4*) shows a correlation with *TP63* of 0.727 when the maximum correlation is 1. The Grasso prostate dataset is comprised of 19574 genes measured in 122 patients, including 28 benign prostate tissue specimens, 59 primary localized prostate carcinoma and 35 metastatic prostate cancer samples. (B) The expression values of *CTEN* and *TP63* in the Grasso prostate dataset are plotted and the graph shows a positive linear correlation. (C) A box-and-whisker plot that represents *TP63* and *CTEN* expression from primary tumors to metastatic lesions in the Grasso prostate dataset. The horizontal line that forms the top and the bottom of each box represents the 75th and the 25th percentile, respectively. The band that intersects the box is the median value and the square represents the mean value. Horizontal lines (whiskers) above and below the box represent maximum and minimum values, respectively. The cross represents an outlier, which is a value greater or lower than the value represented by the top or bottom whisker. (D) The GSE3325 gene expression dataset was analyzed. The expression values of *CTEN* and *ΔNp63* in the dataset are plotted and the graph shows a positive linear correlation. (E) A box-and-whisker plot that represents *ΔNp63* and *CTEN* expression in the GSE3325 dataset obtained from the Gene Expression Omnibus (GEO).

Although p63 protein is highly enriched in the normal prostatic epithelium, it is typically progressively decreased in prostate cancers [48,55]. Similarly, CTEN is abundant in normal prostate but down-regulated in prostatic cancer cells. To explore the clinical correlation between *CTEN* and *p63* expression, we analyzed the Grasso prostate dataset acquired from the Oncomine online microarray database (http://www.oncomine.org). The Grasso prostate dataset is comprised of 122 patients, including 28 benign prostate tissue specimens, 59 primary localized prostate carcinoma and 35 metastatic prostate cancer samples. Coexpression analysis shows a clear positive association between *CTEN* and *p63* mRNA levels with a correlation factor of 0.727 (Fig 1A). The expression values of *CTEN* and *p63* in the Grasso prostate dataset are also plotted and the graph shows a strong positive linear correlation (Fig 1B). The expression of both *CTEN* and *p63* is significantly decreased in prostate cancer specimens compared with that of normal prostate tissues (Fig 1C). Moreover, *CTEN* and *p63* down-regulation correlates with prostate cancer progression from primary tumors to metastatic lesions (Fig 1C). Because ΔNp63 isoforms are the most highly expressed p63 isotypes in the prostate basal epithelial cells, we further examined the expression of *CTEN* and *ΔNp63* in the GSE3325 dataset, which was obtained from the Gene Expression Omnibus (GEO, http://www.ncbi.nlm.nih.gov/geo/). The mRNA levels of *CTEN* and *ΔNp63* in the GSE3325 dataset show a positive association with a correlation factor of 0.657 (Fig 1D) and their down-regulation also correlates with human prostate tumorigenicity and metastasis (Fig 1E).

### ΔNp63α is the predominant p63 isoform in prostate epithelial cells and its expression strikingly correlates with CTEN

**Fig 2:**
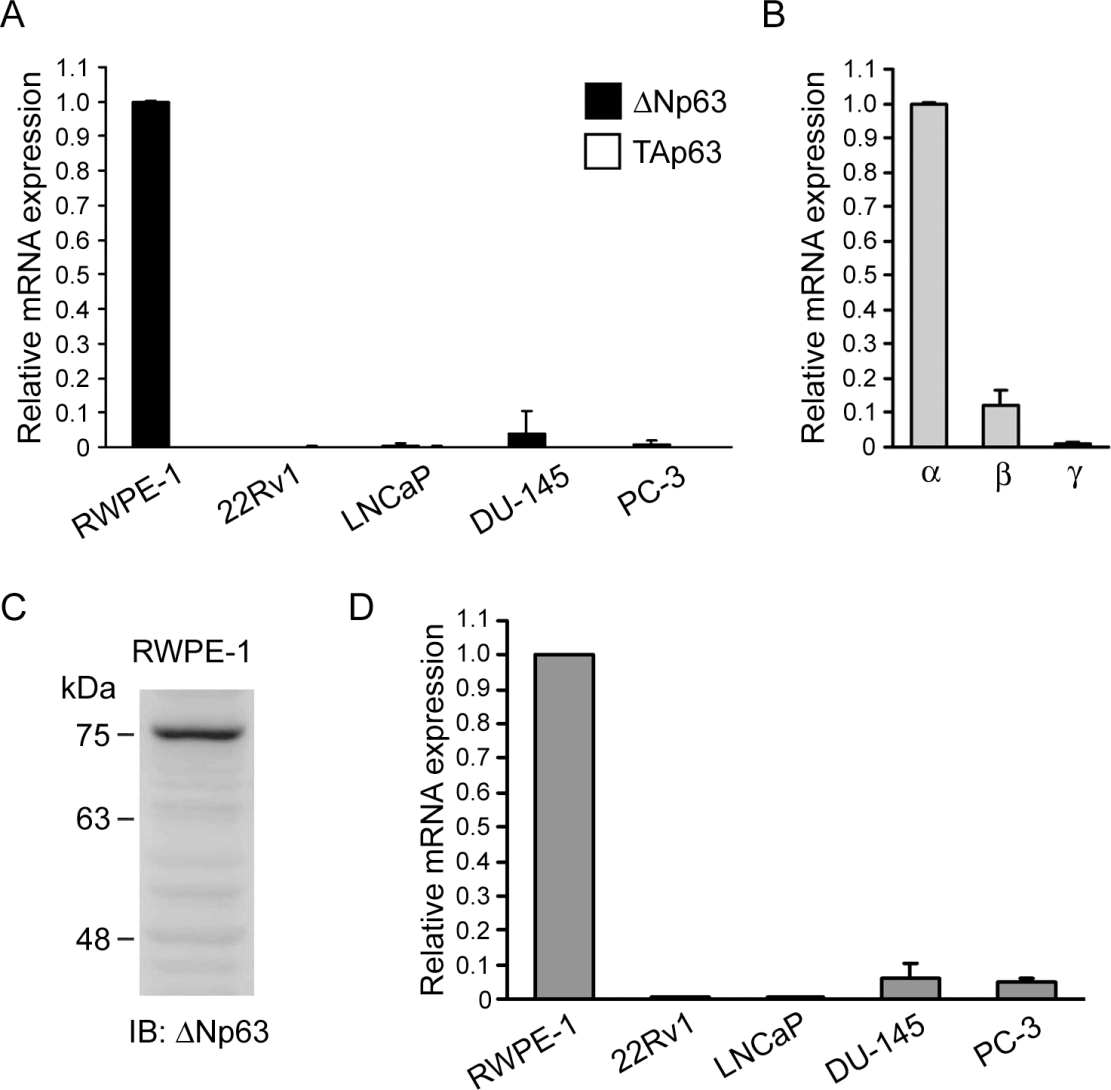
ΔNp63α is the predominant p63 isoform in prostate epithelial cells and its expression strikingly correlates with CTEN. (A) qPCR analyses of the mRNA levels of TAp63 (□) and ΔNp63 (■) isoforms in prostate cell lines. The relative mRNA expression levels were represented as relative fold change compared to the mRNA abundance in RWPE-1 cells. Data are expressed as the mean±standard deviation of three different experiments analyzed in triplicate. (B) qPCR analyses of the mRNA levels of p63α, β and γ isoforms in RWPE-1 cells. The relative mRNA expression levels were represented as relative fold change compared to the mRNA abundance of α isoforms. Data are expressed as the mean±standard deviation of three different experiments analyzed in triplicate. (C) Total lysate (30 μg) of RWPE-1 cells was analyzed by western analysis using antibodies against ΔNp63 isoforms. (D) qPCR analyses of the mRNA levels of CTEN in prostate cell lines. The relative mRNA expression levels were represented as relative fold change compared to the mRNA abundance in RWPE-1 cells. Data are expressed as the mean±standard deviation of three different experiments analyzed in triplicate.

Because most of the p63 probes from online microarray databases hybridize to all p63 isoforms, we next attempted to examine the expression pattern and relative abundance of *CTEN*, *TAp63* and *ΔNp63* in a panel of prostate and cancer cell lines by quantitative real-time PCR (qPCR). In N-terminal p63 isoforms, the mRNA levels of *ΔNp63* are highly abundant in the human nonmalignant prostate epithelial cell line, RWPE-1, whereas those of *TAp63* are nearly undetectable (Fig 2A). In the prostate cancer cell lines, *TAp63* mRNA expression is also barely detected in 22Rv1, LNCaP, DU-145 and PC-3 cells. *ΔNp63* mRNA levels are low in DU-145 and PC-3 cells (3.8% and 0.8% of those in RWPE-1 cells, respectively) and almost undetectable in 22Rv1 and LNCaP cells (Fig 2A). These results suggest that the ΔNp63 variants are much more abundant than TA variants in RWPE-1 cells and these two isoforms are either undetectable or expressed at very low levels in the prostate cancer cell lines we tested. Next, the relative mRNA levels of C-terminal p63 isoforms in RWPE-1 cells were examined by qPCR and we demonstrated that the p63α isoforms are much more enriched than p63β and p63γ isoforms (Fig 2B). In addition, we also used an antibody that specifically recognizes the amino acids 5–17 of the N-terminal domain of ΔNp63 to identify the expression patterns of this isoform in RWPE-1 cells. The theoretical molecular weight of ΔNp63α, ΔNp63β and ΔNp63γ is 66, 51 and 45 kDa, respectively, but their apparent molecular weight is higher than predicted [56]. We found that ΔNp63α is the major isotype as detected by immunoblotting in RWPE-1 cells, indicating that ΔNp63α is the predominant p63 isoform in normal prostate cells (Fig 2C). Moreover, the mRNA expression pattern of *CTEN* in the prostate cell lines shows a similar trend to that of *ΔNp63* (Fig 2D). *CTEN* transcripts are markedly higher in RWPE-1 cells but are significantly lower in DU-145 and PC-3 cells with 17-fold and 21-fold decreases, respectively, and at least 200 times lower in 22Rv1 and LNCaP cells.

As ΔNp63 isoforms, particularly the ΔNp63α splice variant, are expressed predominantly in prostate cells, our data indicates that the expression of CTEN strikingly correlates with that of ΔNp63α in human benign prostate tissues and carcinomas. This observation raises the possibility that ΔNp63α transcriptionally regulates CTEN and that dysfunction of the ΔNp63α-CTEN regulatory axis potentially contributes to tumorigenesis.

### The expression of CTEN is transcriptionally regulated by ΔNp63α

**Fig 3:**
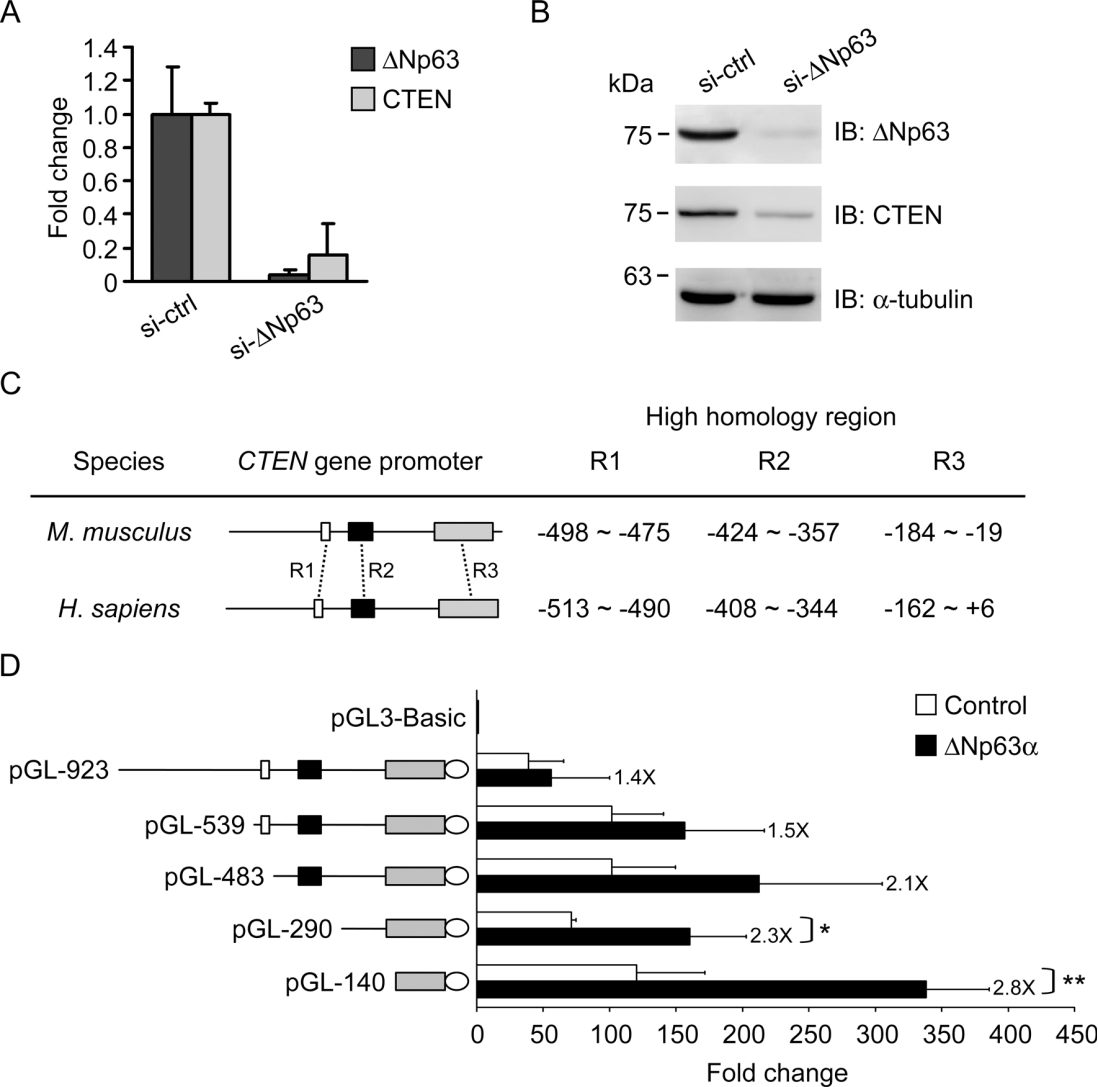
*CTEN* is transcriptionally regulated by ΔNp63α. RWPE-1 cells were transfected by control siRNA (si-ctrl) or ΔNp63 siRNA (si-ΔNp63). qPCR (A) and western (B) analyses of ΔNp63 and CTEN expression were performed 48 hr after transfection. α-tubulin in western analysis was used as a loading control. (C) Schematic representation of the CTEN promoter region in mouse and human. Three regions of high homology were identified and are shown as R1 (white boxes), R2 (black boxes) and R3 (grey boxes). (D) The serial deletions at the 5’-end of the *CTEN* promoter are schematically shown on the left. Different lengths of the *CTEN* promoter were subcloned into the pGL3-Basic reporter vector (designated pGL), and the numbers represent the position relative to the transcription initiation site. Non-coding exon 1 is shown in ellipse. Each chimeric reporter construct was cotransfected with a ΔNp63α expressing plasmid (■) or with an empty vector (□) into HEK293 cells. In addition, the pRL-TK vector, which contains the *Renilla* luciferase reporter gene, was also cotransfected as an internal control. Dual luciferase assays were performed 48 hr after transfection and the individual relative light units (RLU) from the firefly luciferase (*CTEN* promoter activity) were normalized to that from the *Renilla* luciferase. The final value is expressed as the mean relative fold change compared to RLU from pGL3-Basic for each fragment. Data are expressed as the mean±standard deviation of four different experiments analyzed in triplicate. Numbers next to the error bars indicate the fold induction of activity in ΔNp63α-expressed cells compared to that in the empty vector-transfected cells. (*: P <0.05, **: P <0.001)

To investigate whether the association between ΔNp63α and CTEN expression is due to their functional relationship in the prostate, small interfering RNA (siRNA)-mediated gene silencing was first used to determine the effect of ΔNp63α depletion on CTEN expression. ΔNp63 mRNA and protein expression are markedly reduced by siRNA selectively against ΔNp63 in RWPE-1 cells and depletion of ΔNp63α diminished the accumulation of CTEN mRNA and protein (Fig 3A and 3B). This suggests that CTEN expression is controlled by ΔNp63α and ΔNp63α might directly act on the *CTEN* promoter to regulate its expression at the transcriptional level.

To assess whether *CTEN* is a direct transcriptional target of ΔNp63α, luciferase reporter assays were performed. We first compared the sequences of human and mouse *CTEN* promoter and found three regions of high homology (designated R1, R2 and R3) as shown in Fig 3C. To analyze the promoter activity, approximately 1 kb upstream of the transcription initiation site (designated +1) of the human *CTEN* gene together with the non-coding exon 1 was PCR amplified using genomic DNA from RWPE-1 cells as a template. After sequence verification, the fragment (-923 to +40) was subcloned into the pGL3-Basic vector, which contains the luciferase reporter gene but lacks a eukaryotic promoter or enhancer sequences, resulting in the pGL-923 plasmid (Fig 3D). Serial deletions of the *CTEN* promoter region subcloned into the pGL-Basic reporter plasmid were also generated as described in materials and methods. Each chimeric reporter construct was then cotransfected with a ΔNp63α expressing plasmid or an empty vector into HEK293 cells. In addition, the pRL-TK vector, which contains the *renilla* luciferase reporter gene, was also cotransfected to monitor the transfection efficiency.

As shown in Fig 3D, the 923-bp *CTEN* promoter region (pGL-923) is capable of driving luciferase expression and its activity is enhanced by ΔNp63α with a 1.4-fold induction. Deletion of the region from -923 to -540 (pGL-539) retains the capability to drive expression and leads in a 1.5-fold increase in the presence of ΔNp63α. When the conserved R1 and R2 are further deleted (pGL-483 and pGL-290), ΔNp63α results in 2.1- and 2.3-fold activation, respectively. A more significant up-regulation (2.8-fold) by ΔNp63α is observed in pGL-140, which contains the partially conserved R3. These results demonstrate that *CTEN* is transcriptionally activated by ΔNp63α and that the 140-bp fragment upstream of the transcription initiation site is the minimal promoter region required for activation.

Although the major p63 isoform expressed in prostate cells is ΔNp63α, we can not exclude the possibility that other p63 isoforms could regulate the transcriptional activity of *CTEN* promoter. Therefore, we further overexpressed different isoforms of p63 (ΔNp63α, ΔNp63β, ΔNp63γ, TAp63α) in HEK293 cells and monitored their effect on the reporter activity of pGL-140 (S1 Fig). A significant transcriptional activation of *CTEN* promoter (2.2 fold) is observed only in the ΔNp63α-expressed cells (S1B Fig). However, HEK293 cells might lack cofactors required for the p63-mediated activation of *CTEN* gene expression because the endogenous expressions of ΔNp63α and CTEN are not detectable in HEK293 cells. We thus also evaluated the effects of expressing p63 isoforms on the promoter activity and the endogenous mRNA levels of *CTEN* in RWPE-1 cells (S2 Fig). Consistent with results in HEK293 cells, TAp63α failed to enhance pGL-140 reporter gene expression in RWPE-1 cells, presumably as a result of differential transactivation activity arising from the amino terminus (S1B and S2B Figs). Moreover, ΔNp63β and ΔNp63γ, which did not transactivate the pGL-140 reporter in HEK293 cells, were able to induce *CTEN* promoter activity with 1.7-fold and 2.3-fold increases, respectively (S1B and S2B Figs). Overexpression of ΔNp63α resulted in a markedly higher induction of *CTEN* promoter activity (9.0 fold) in RWPE-1 cells, suggesting that the C-terminal domains specifically present in α isotype are important for its transactivation activity on *CTEN* promoter. However, the endogenous mRNA levels of *CTEN* in RWPE-1 cells were not increased by the exogenously expressed p63 isoforms (S2C Fig). This might result from a saturation effect of p63-regulatory machinery on endogenous CTEN expression due to ΔNp63α and CTEN are both highly abundant in RWPE-1 cells.

### *CTEN* promoter contains a p63-binding site and ΔNp63α directly interacts with *CTEN* promoter

**Fig 4:**
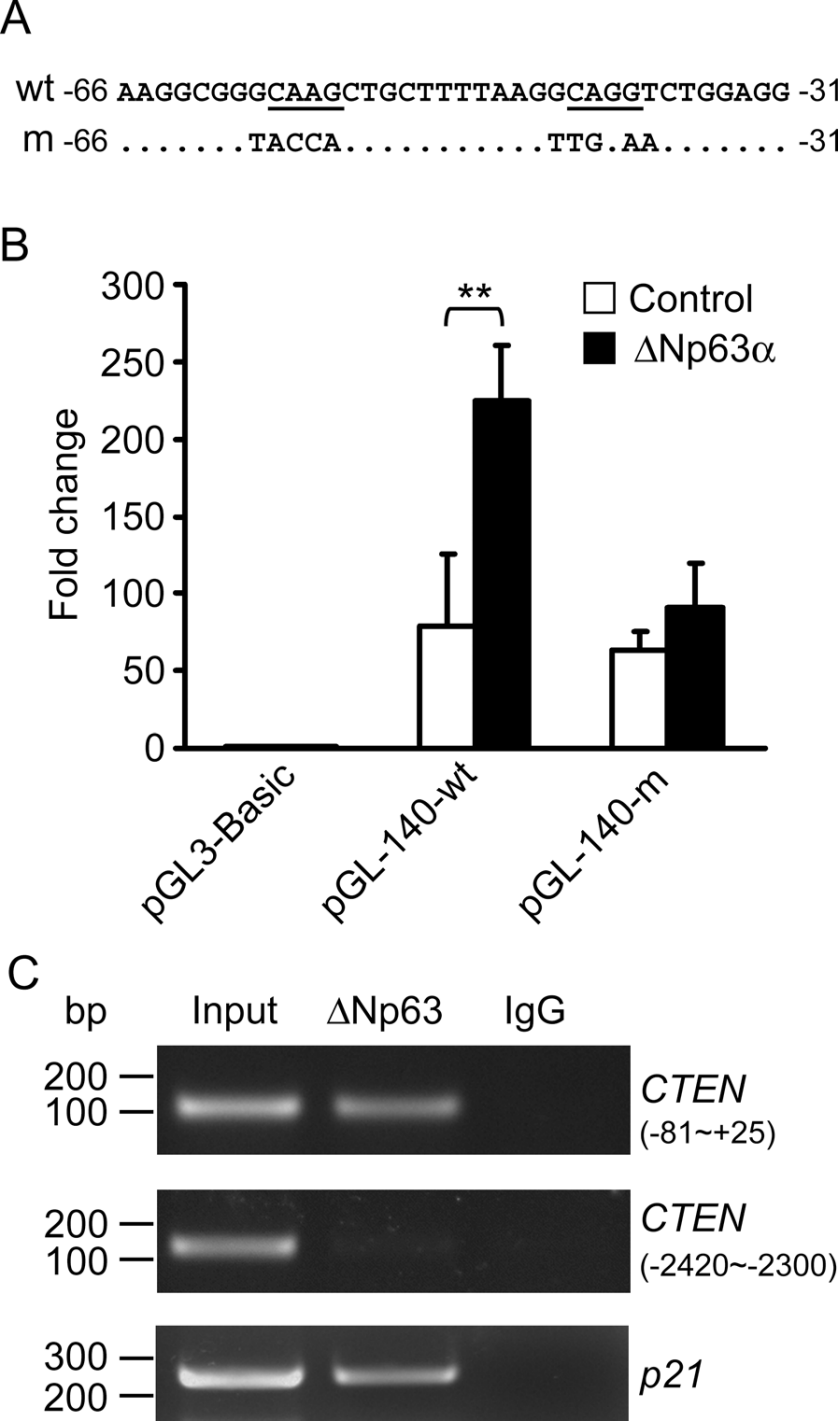
*CTEN* promoter contains the p63-binding regulatory sequences and ΔNp63α directly binds to the *CTEN* promoter. (A) Analysis of the *CTEN* promoter identified putative binding sites for p63 located between -61 and -36. DNA sequences around this region are shown as “wt” and the consensus binding sequences are underline. Mutations on the consensus nucleotides are presented as “m”. (B) The wild-type (pGL-140-wt) or mutant pGL-140 (pGL-140-m) CTEN promoter construct was cotransfected with a ΔNp63α expressing plasmid (■) or with an empty vector (□) into HEK293 cells. In addition, the pRL-TK vector was also cotransfected as an internal control. Dual luciferase assays were performed 48 hr after transfection and the *CTEN* promoter activity is presented as the ratio of firefly/*Renilla* luciferase activity. Data are expressed as the mean±standard deviation of three different experiments analyzed in triplicate. (**: P <0.005) (C) Chromatin extracted from RWPE-1 cells was immunoprecipitated with anti-ΔNp63 antibodies or rabbit IgG. The DNA fragments were released and amplified by PCR using *CTEN* promoter-specific primers that recognize the p63 binding region (-81 to +25) or an upstream unrelated region (-2420 to -2300). The *p21* promoter-specific primers were used as a positive control.

Because the above results suggest that the *CTEN* promoter might contain p63-binding regulatory sequences, we next performed an *in silico* analysis to identify putative p63 responsive elements in the *CTEN* promoter region. Sequence analysis by the TRANSFAC database revealed the presence of a putative binding site for p63 located between -61 and -36 within the *CTEN* promoter (Fig 4A, wt). To further assess the ΔNp63α responsive region, we generated a plasmid with mutations in the critical nucleotides on the putative binding site within the 140-bp promoter using site-directed mutagenesis (Fig 4A, m). These mutations abrogate p63 binding and significantly diminish the promoter activity induced by ΔNp63α (pGL-140-m, Fig 4B). It suggests that the putative binding site is indeed a critical element for ΔNp63α activation.

To verify the direct binding of ΔNp63α to the *CTEN* promoter, we subsequently performed a chromatin immunoprecipitation (ChIP) assays. Chromatin extracted from RWPE-1 cells was immunoprecipitated with an anti-ΔNp63 antibody, and the immunoprecipitated chromatin was subjected to PCR. Specific primers for the *CTEN* promoter region comprising the putative p63-binding site (-81 ~ +25) and an upstream unrelated region (-2420 ~ -2300) were used. Primers specifically annealing to the *p21* promoter were used as a positive control [57]. As shown in Fig 4C, the *CTEN* specific amplicons from -81 to +25 are detected only in the anti-ΔNp63 immunoprecipitated chromatin, but no specific PCR band is present in the IgG control. The promoter region from -2420 to -2300 with no p63-binding site shows negative for ΔNp63 binding. Therefore, the ChIP assay demonstrates that ΔNp63 directly interacts with the *CTEN* promoter. Because ΔNp63α is the major p63 isoform endogenously expressed in RWPE-1 cells, these data strongly suggest that ΔNp63α physically binds to the *CTEN* promoter to transactivate its expression and that the sequences from -61 to -36 mediate this positive regulatory function.

When we examined the p63 ChIP-seq data from EP156T prostate cells, human primary keratinocytes, human primary foreskin keratinocytes and HaCaT keratinocytes, we found CTEN is identified as a p63 target gene as well in all these studies [58–62]. ChIP-seq, a combination of chromatin immunoprecipitation (ChIP) assays with sequencing, is a powerful and widely used approach for mapping the genome-wide DNA binding sites for transcription factors and other proteins. The p63 ChIP-seq peaks in these reports were mapped to the human genome NCBI build 37 and we summarized the genome-wide p63-binding sites associated with the *CTEN* locus in S2 Table. The majority of p63 peaks appearing within 50 kb from the transcription start site (TSS) of *CTEN* gene are located in intron 2 as well as in 8.9 kb and 36.6 kb regions upstream of the TSS. However, the binding sequences they identified do not contain the binding site we analyzed, which located between -61 and -36 within the *CTEN* promoter. These p63 binding sites associated with *CTEN* from ChIP-seq studies were verified by qPCR following ChIP using specific primers for the *CTEN*-associated regions comprising ChIP peaks listed in S1 Table. ChIP-qPCR values were first normalized by the respective input values and then fold enrichments were calculated compared with enrichment of the *CTEN* promoter region not expected to interact with ΔNp63α (-2420 ~ -2300). ChIP-qPCR results demonstrate ΔNp63α binds to the target sites identified by ChIP-seq and our study (S3 Fig). However, ΔNp63α occupancy exhibited a higher enrichment for the regions far upstream of *CTEN* TSS (-36.6 and -8.9 kb to TSS) but lower enrichment of ΔNp63α binding was detected at the intron2 region (7.6 kb and 11 kb to TSS) as well as the binding site we analyzed (-0.1 kb to TSS) (S3 Fig).

### Regulation of CTEN by ΔNp63α is associated with cell adhesion

**Fig 5:**
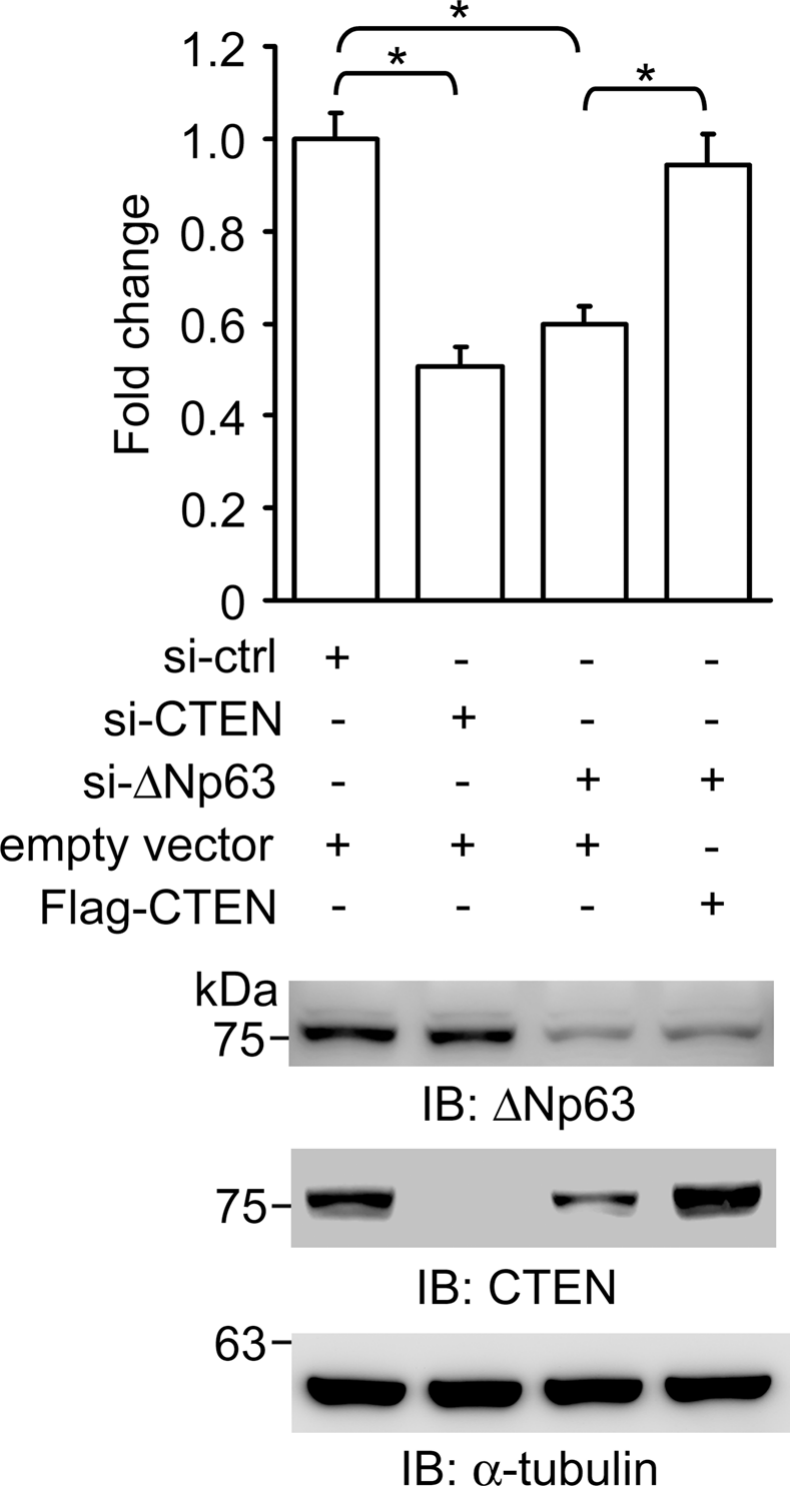
Expression of CTEN rescues impaired cell adhesion caused by ΔNp63 deficiency. RWPE-1 cells were transfected by control siRNA (si-ctrl), CTEN-specific siRNA (si-CTEN) or ΔNp63-specific siRNA (si-ΔNp63) together with FLAG-tagged CTEN expression plasmids (Flag-CTEN) or the corresponding empty vector. Cells were reseeded on plates coated with fibronectin 48 hr after transfection. Non-adherent cells were washed off by PBS and adherent cells were stained, solubilized and quantified by measuring the absorbance at 595 nm as described in materials and methods. The absorbance from each sample was normalized to that from the control (si-ctrl +; empty vector +) (upper panel). The final value is expressed as the mean relative fold change compared to the control. Data are expressed as the mean±standard deviation of three different experiments analyzed in triplicate. (*: P <0.005) Western analyses of ΔNp63 and CTEN expression were performed 48 hr after transfection. α-tubulin was used as a loading control. (lower panel)

ΔNp63α functions as a key regulator of epithelial cell adhesion. Loss of ΔNp63α has previously been shown to cause decreased binding to the extracellular matrix (ECM) in epithelial cells [30,58]. It has been documented that cancer cells must develop altered affinity for their ECM, resulting in dissociation from the origin, to initiate the metastatic process. We therefore asked whether CTEN acts as a downstream mediator of ΔNp63α function in regulating cell adhesion in RWPE-1 cells. The functional consequences of changes in CTEN expression levels on cell adhesion were evaluated by examining the ability of cells to adhere to fibronectin, an ECM protein. CTEN is effectively silenced by *CTEN*-specific siRNA and knockdown of *CTEN* suppresses cell adhesion approximately 50% (Fig 5). In addition, ablation of ΔNp63α by siRNA in RWPE-1 cells resulted in down-regulation of CTEN and this also significantly reduces the ability of cell adhesion (Fig 5 and S4 Fig). Similar results were also obtained using collagen I as an exogenous matrix protein (data not shown). Furthermore, ectopic expression of CTEN rescues the impaired cell adhesion caused by ΔNp63 depletion, suggesting that CTEN is a downstream effector of ΔNp63-mediated cell adhesion (Fig 5).

## Discussion

Because p63 functions as an important transcriptional regulator and is required for normal prostate development, characterization of its expression pattern and downstream targets is critical for deciphering the function of p63 in prostate. Our study sheds light on a novel correlation between ΔNp63α and CTEN expression in prostate tumor and normal cell lines and tissues. We report for the first time that ΔNp63α activates transcription of *CTEN* by directly binding the *CTEN* promoter. Our work highlights CTEN as a novel ΔNp63α target for the regulation of cell adhesion and indicates that CTEN is associated with prostate cancer progression.

Our study shows that ΔNp63α is the most abundant p63 isoform expressed in normal prostate epithelial cells and is down-regulated in prostate cancer (Figs 1 and 2), a finding consistent with previous reports [37,63]. Our expression analyses in prostate cell lines and tissues indicate that the expression of CTEN has a similar distribution and is strikingly correlated with ΔNp63α (Figs 1 and 2). The expression of *CTEN* is significantly decreased in prostate cancer and is correlated with prostate cancer progression from primary tumors to metastatic lesions (Fig 1). ΔNp63α knockdown results in a marked reduction in CTEN expression (Fig 3A and 3B) and a significant decrease in cell adhesion (Fig 5) in the RWPE-1 prostate epithelial cell line. In addition, over-expression of CTEN in ΔNp63α-depleted RWPE-1 cells rescues the impaired cell adhesion (Fig 5). These data indicate that CTEN is a downstream effector of ΔNp63α-mediated cell adhesion. It has been demonstrated that p63 plays a critical role in the regulation of epithelial cell adhesion. In a model of human primary prostate cells, EP165T, ΔNp63α is lost during epithelial to mesenchymal transition (EMT) and its re-expression in the mesenchymal counterpart, EPT1, led to gain of several epithelial characteristics [58,64]. When ΔNp63α is knocked down, EP165T exhibits significantly reduced binding to fibronectin, laminin and collagen IV [58]. In a previous study, RNAi against p63 in RWPE-1 cells also resulted in the acquisition of a luminal phenotype and loss of epithelial adhesion [65]. In addition to prostate cells, p63 is also a critical regulator of cellular adhesion in keratinocytes and mammary epithelial cells [30,66]. These findings, together with our result that ΔNp63α depletion impairs cell adhesion (Fig 5), indicate that ΔNp63α regulates a cell adhesion-related gene network. Several molecules, such as integrin β4, α6 and laminin-γ2, have been identified as ΔNp63α downstream regulators in cell adhesion [30,58]. Although CTEN has been identified as a ΔNp63α downstream gene by analyzing differentially expressed genes in Seborrheic keratosis [49], we report for the first time that CTEN is a ΔNp63α target in regulation of prostate cell adhesion. CTEN loss in prostate epithelial cells is associated with decreased cell adhesion, which might contribute to losing the epithelial characteristics and acquiring the mesenchymal-like traits. Given that cells might accumulate malignant features during EMT, in which they initially decrease their binding to the ECM, we assume that the dysfunction of the ΔNp63α-CTEN regulatory axis potentially contributes to tumorigenesis in the prostate. We are currently addressing these possibilities.

CTEN has several unique features among tensin members. The molecular mass of CTEN is about half of other members and its protein structure only shares the conserved C-terminal SH2 and PTB domains. Moreover, CTEN expression pattern is much more restricted than for other tensins [50]. CTEN appears to be a newly evolved gene that is highly expressed in the prostate and is frequently down-regulated in human prostate cancer and benign prostatic hyperplasia [52]. However, the mechanisms regulating CTEN expression remain largely unknown. We identified three regions (R1, R2 and R3) of high sequence homology between the human and mouse *CTEN* promoter. On the basis of our results of the promoter deletion analysis, we demonstrate that ΔNp63α enhances the transcriptional activity of every *CTEN* promoter region tested ranging from 1.4- to 2.8-fold (Fig 3D). The activation is more pronounced in the pGL-140 construct suggests that the promoter region upstream of R3 might contain binding sites for some transcription repressors, which could interact with ΔNp63α to attenuate ΔNp63α-mediated transactivation. We further determine that the 140-bp fragment upstream of the transcription initiation site is not only the minimal promoter region for directing expression of *CTEN* but also contains ΔNp63α-binding regulatory sequences. Sequence analysis by the TRANSFAC database identified a putative binding site for p63 within the R3 region that was demonstrated to be a critical element for ΔNp63α binding and activation (Fig 4). The *CTEN* promoter has significantly strong activity in human prostatic epithelial cell lines compared to other cell types in reporter assays [52]. Two potential prostate-specific transcription factor (PSE) binding sites located on the CTEN promoter, however, do not contribute to its preferential promoter activity in the prostate [52]. Because ΔNp63α plays a critical role in prostate epithelial development and activates CTEN expression in the prostate, our study suggests that ΔNp63α might function as a trans-regulatory element mediating the expression of CTEN in the prostate.

Although the major p63 isoform expressed in prostate cells is ΔNp63α, other p63 isoforms present in different cell types might be able to regulate the transcriptional activity of *CTEN* promoter. To determine whether ΔNp63α is unique among the p63 isoforms in its transactivation activity on *CTEN*, the effects of different p63 isoforms on *CTEN* promoter activity were examined both in HEK293 and RWPE-1 cells (S1 and S2 Figs). ΔNp63α resulted in the highest induction of *CTEN* promoter activity among the p63 isoforms whereas TAp63α failed to enhance *CTEN* reporter gene expression in both cell lines, suggesting that the amino terminus of TA and ΔN isoforms possesses differential transactivation activity (S1B and S2B Figs). It has been reported that the amino terminal 26 amino acids of ΔNp63 encode a novel transactivation domain [9,11], in which 14 amino acids are unique to the ΔNp63 isoforms and 12 amino acids are common to both TA and ΔN isoforms. Thus, the ΔNp63-specific amino acids within the amino terminus may be critical for *CTEN* transcriptional control. On the other hand, ΔNp63β and ΔNp63γ isoforms induce *CTEN* promoter activity in RWPE-1 cells but not in HEK293 cells, suggesting that additional cofactors not available in HEK293 may be required for their transactivation activities. Although α, β and γ ΔNp63 isotypes all stimulated *CTEN* promoter activity in RWPE-1 cells, only ΔNp63α exhibited the strongest induction, suggesting that C-terminal SAM protein-protein interaction domain specific to α splice variants of p63 may be essential for transcriptional coactivator recruitment.

The p63-binding motif on the *CTEN* promoter (Fig 4A) is very similar to those previously described [58,61,67]. When we examined the genome-wide profile of p63 binding targets in EP156T cells [58], we found CTEN is one of the 2,487 targets they identified using ChIP-seq. After functional annotations, CTEN is one of 82 genes related to the cytoskeletal protein binding containing p63 binding sites. When ΔNp63α is ectopically expressed in EPT1 cells (which are derived from EP165T following EMT), CTEN re-expression is induced by ΔNp63α. However, the binding sequences they identified do not contain the p63 binding site we analyzed on *CTEN* promoter (-61 to -36). We also examined p63 ChIP-seq data from human primary keratinocytes [59,68], primary human foreskin keratinocytes [60,61] and a human keratinocyte cell line HaCaT [62]. CTEN is also identified as a p63 target gene in all the studies but the p63-binding site on proximal *CTEN* promoter that we analyzed is not overlapped with the p63-binding regions they mapped. This may be due to ChIP-seq variations resulted from binding affinity, specificity and stability between protein and DNA as well as ChIP-antibody quality and alignment algorithm. The majority of p63 peaks identified by ChIP-seq are located in intron 2 (7.6 and 11 kb) kb as well as in 8.9 kb and 36.6 kb regions far upstream of the TSS, which we did not analyze in our proximal *CTEN* promoter-reporter constructs. These p63 binding sites associated with *CTEN* identified by ChIP-seq and our study were verified by qPCR following ChIP. We demonstrated that ΔNp63α binds to all the target sites we tested but ΔNp63α occupancy exhibited relatively lower enrichment on the binding site we identified (-0.1 kb to TSS) (S3 Fig). Further studies need to be conducted to determine their functions on *CTEN* transcriptional regulation because transcriptional regulators may modulate from a distance of thousands of base pairs away from the initiation site by looping DNA to bring the regulator complex close to the core promoter. Nonetheless, our current studies do not exclude the possibility that the transcriptional regulatory mechanism of *CTEN* gene might be distinct in different cell or tissue types. Epigenetic modifications or transcription regulators preferentially expressed in specific cells or tissues may contribute to the control of *CTEN* expression.

In conclusion, our findings provide a better understanding on the molecular regulation of the *CTEN* gene. We explore the role of CTEN in the regulation of prostate cell adhesion and in association with prostate cancer progression. Our findings indicate that dysfunction of the ΔNp63α-CTEN regulatory axis potentially contributes to prostate tumorigenesis.

## Supporting Information

S1 Fig Effects of p63 isoforms on the promoter activity of *CTEN* in HEK293 cells. The pGL-140 *CTEN* promoter construct was cotransfected with ΔNp63α, ΔNp63β, ΔNp63γ, TAp63α expressing plasmids or an empty vector (control) into HEK293 cells. (A) 48 hr after transfection, total cell lysate (20 μg) was analyzed by western analyses. α-tubulin in western analysis was used as a loading control. (B) Dual luciferase assays were performed 48 hr after transfection and the *CTEN* promoter activity is presented as the ratio of firefly/*Renilla* luciferase activity. Data are expressed as the mean±standard deviation of three different experiments analyzed in triplicate. (*: P <0.002 compared with control)
(TIF)

S2 Fig Effects of p63 isoforms on the promoter activity and mRNA levels of *CTEN* in RWPE-1 cells. The pGL-140 *CTEN* promoter construct was cotransfected with ΔNp63α, ΔNp63β, ΔNp63γ, TAp63α expressing plasmids or an empty vector (control) into RWPE-1 cells. (A) 48 hr after transfection, total cell lysate (20 μg) was analyzed by western analyses. α-tubulin in western analysis was used as a loading control. (B) Dual luciferase assays were performed 48 hr after transfection and the *CTEN* promoter activity is presented as the ratio of firefly/*Renilla* luciferase activity. Data are expressed as the mean±standard deviation of three different experiments analyzed in triplicate. (*: P <0.01; **: P<0.005 compared with control) (C) Quantification of endogenous *CTEN* transcripts in RWPE-1 cells transfected by p63 isoforms was analyzed by qPCR as described in materials and methods.
(TIF)

S3 Fig ChIP-qPCR analysis of ΔNp63α enrichment at the *CTEN* locus. ChIP-qPCR values were first normalized by the respective input values and then fold enrichments were calculated compared with enrichment of a *CTEN* promoter region not expected to interact with ΔNp63α (-2420 ~ -2300). *p21* promoter region was used as a positive control. Results are presented as fold enrichment relative to input DNA and the negative control (-2.4 kb). Data are expressed as the mean±standard deviation of three different experiments. (*: P <0.05; **: P<0.01 compared with the negative control)
(TIF)

S4 Fig Knockdown of ΔNp63α results in CTEN down-regulation. Quantification of *ΔNp63* and *CTEN* transcripts in RWPE-1 cells transfected by control siRNA (si-ctrl) or ΔNp63 siRNA (si-ΔNp63) was analyzed by qPCR as described in materials and methods.
(TIF)

S1 Table Primers for qPCR and ChIP-qPCR. (DOC)

S2 Table p63 ChIP-seq peaks associated with the *CTEN* locus identified by previous studies. (DOC)
