# Supplementary figures and images for: ΔNp63α Transcriptionally Regulates the Expression of CTEN That Is Associated with Prostate Cell Adhesion
Source: PLoS One. 2016 Jan 19;11(1):e0147542. doi: 10.1371/journal.pone.0147542 (PMC4718700; doi:10.1371/journal.pone.0147542)

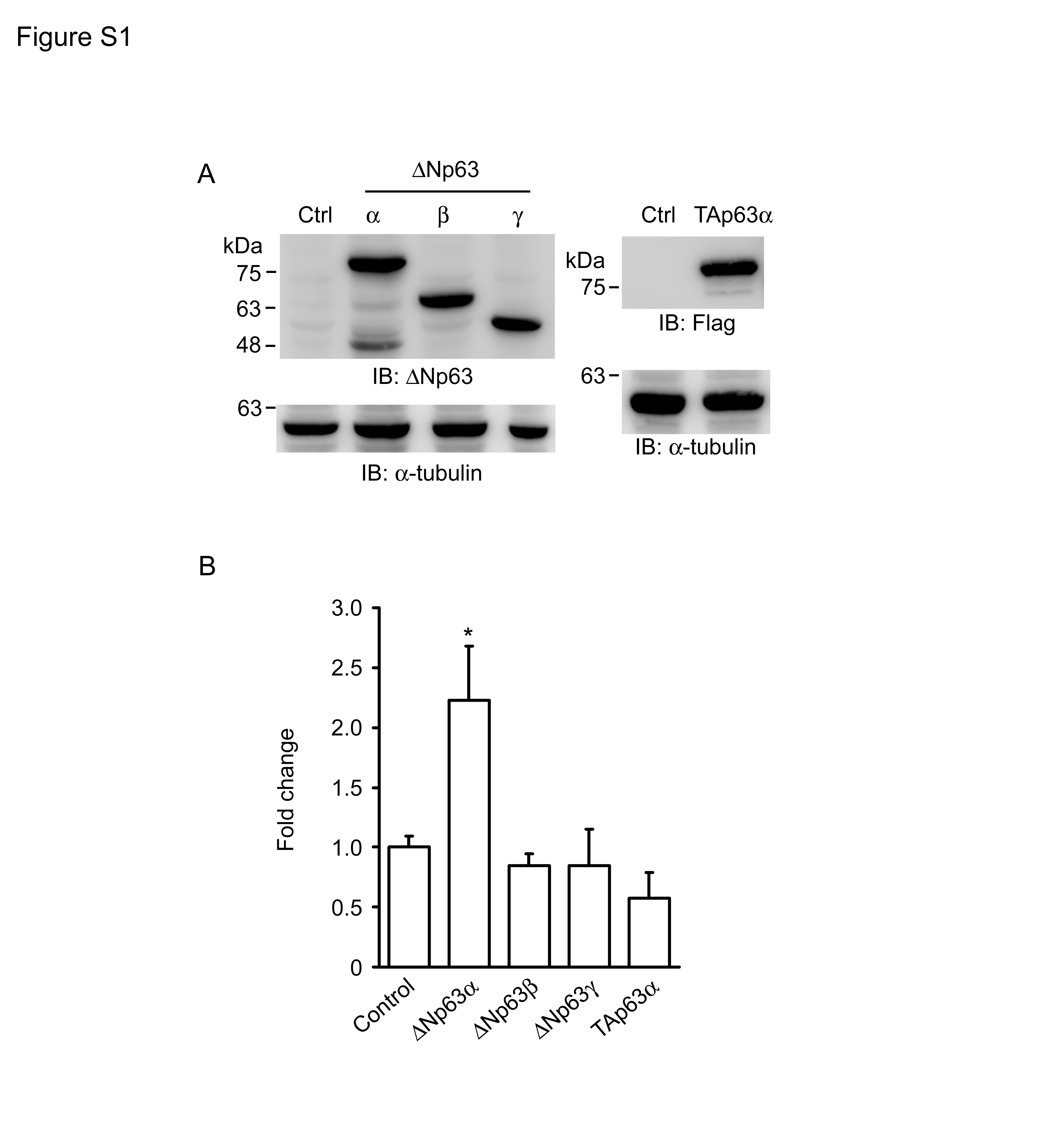

Supplement: S1 Fig — The pGL-140 CTEN promoter construct was cotransfected with ΔNp63α, ΔNp63β, ΔNp63γ, TAp63α expressing plasmids or an empty vector (control) into HEK293 cells. (A) 48 hr after transfection, total cell lysate (20 μg) was analyzed by western analyses. α-tubulin in western analysis was used as a loading control. (B) Dual luciferase assays were performed 48 hr after transfection and the CTEN promoter activity is presented as the ratio of firefly/Renilla luciferase activity. Data are expressed as the mean±standard deviation of three different experiments analyzed in triplicate. (*: P <0.002 compared with control) (TIF) [file pone.0147542.s001.tif]

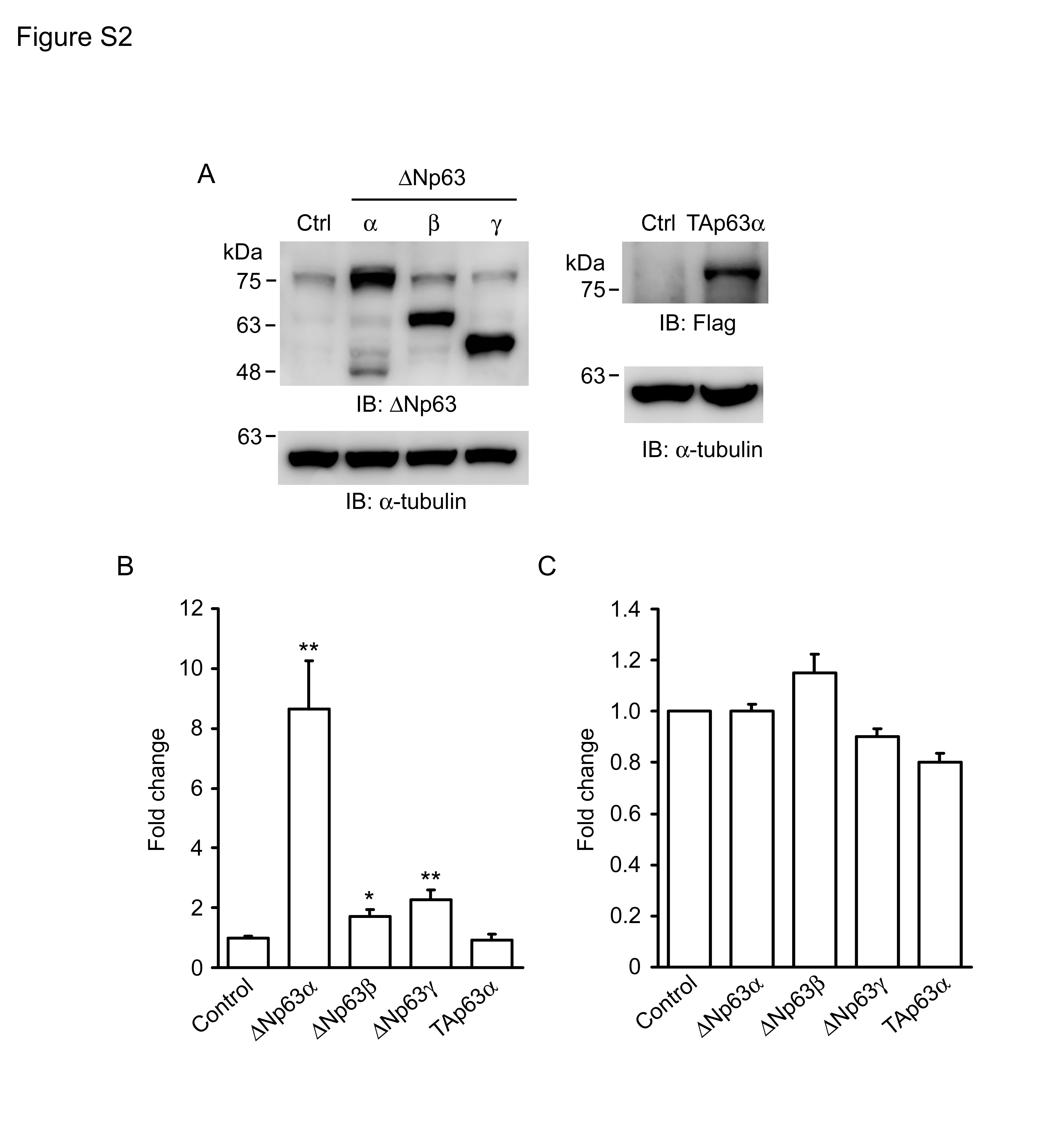

Supplement: S2 Fig — The pGL-140 CTEN promoter construct was cotransfected with ΔNp63α, ΔNp63β, ΔNp63γ, TAp63α expressing plasmids or an empty vector (control) into RWPE-1 cells. (A) 48 hr after transfection, total cell lysate (20 μg) was analyzed by western analyses. α-tubulin in western analysis was used as a loading control. (B) Dual luciferase assays were performed 48 hr after transfection and the CTEN promoter activity is presented as the ratio of firefly/Renilla luciferase activity. Data are expressed as the mean±standard deviation of three different experiments analyzed in triplicate. (*: P <0.01; **: P<0.005 compared with control) (C) Quantification of endogenous CTEN transcripts in RWPE-1 cells transfected by p63 isoforms was analyzed by qPCR as described in materials and methods. (TIF) [file pone.0147542.s002.tif]

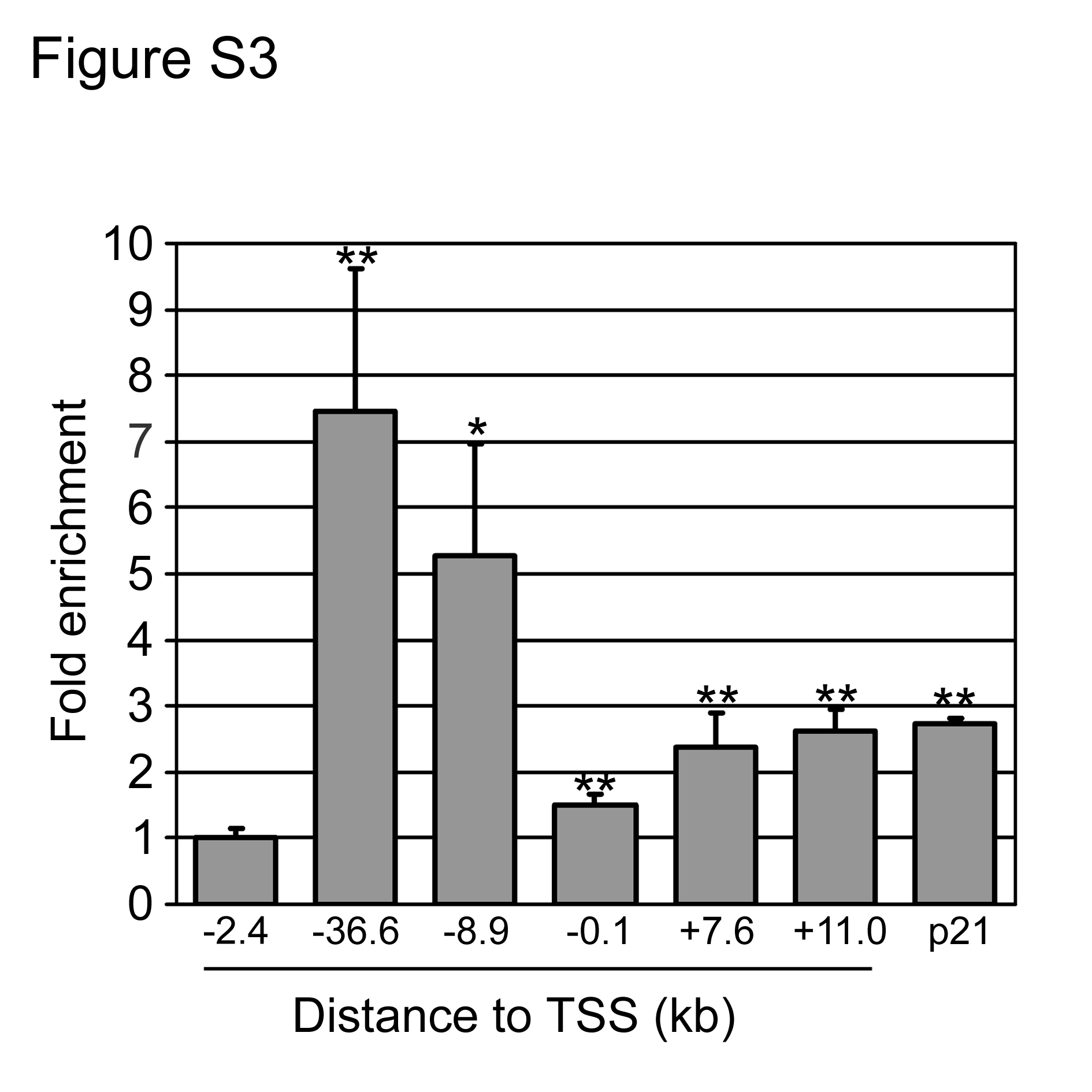

Supplement: S3 Fig — ChIP-qPCR values were first normalized by the respective input values and then fold enrichments were calculated compared with enrichment of a CTEN promoter region not expected to interact with ΔNp63α (-2420 ~ -2300). p21 promoter region was used as a positive control. Results are presented as fold enrichment relative to input DNA and the negative control (-2.4 kb). Data are expressed as the mean±standard deviation of three different experiments. (*: P <0.05; **: P<0.01 compared with the negative control) (TIF) [file pone.0147542.s003.tif]

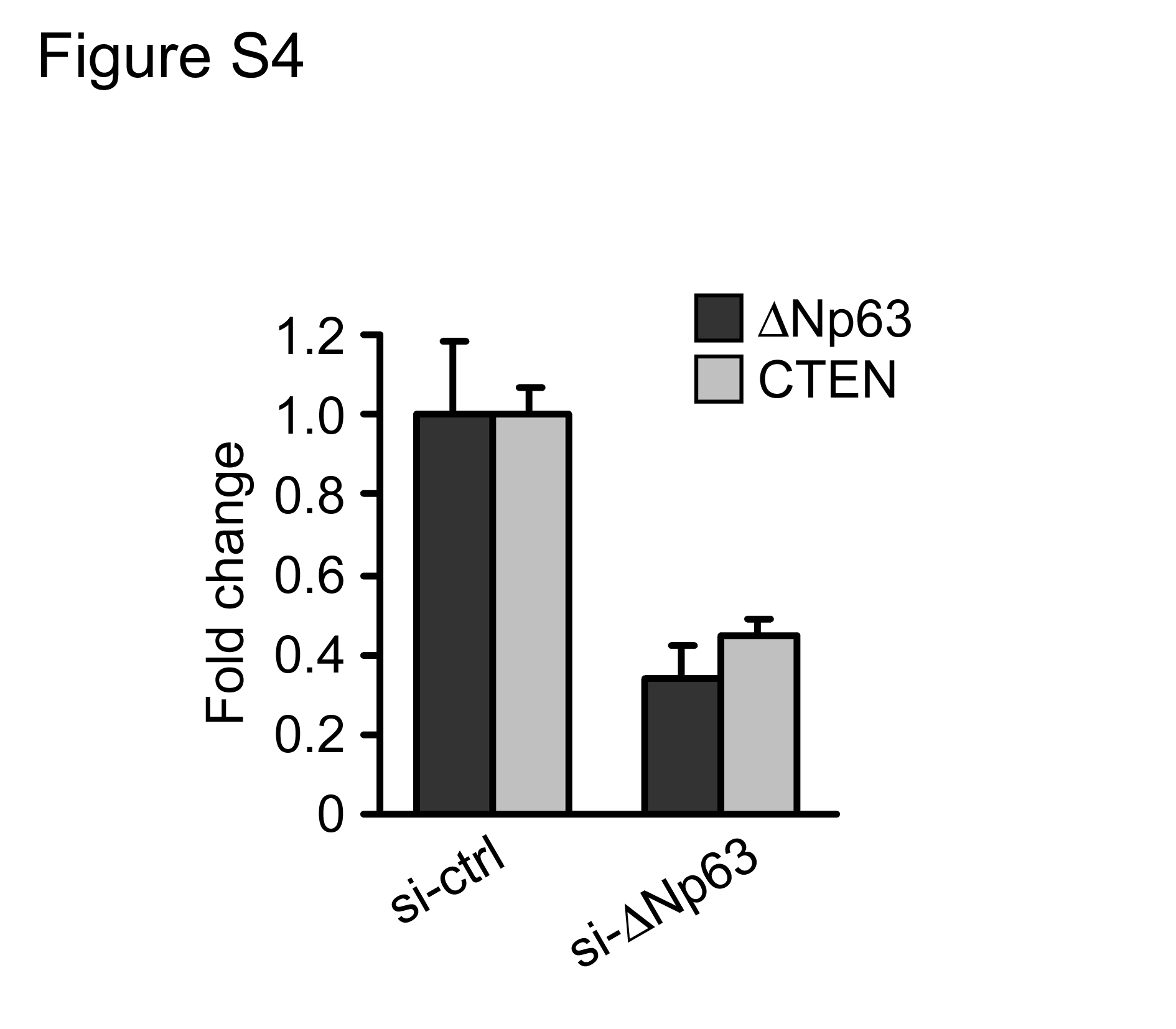

Supplement: S4 Fig — Quantification of ΔNp63 and CTEN transcripts in RWPE-1 cells transfected by control siRNA (si-ctrl) or ΔNp63 siRNA (si-ΔNp63) was analyzed by qPCR as described in materials and methods. (TIF) [file pone.0147542.s004.tif]
