# Supplementary material for: ΔNp63α Transcriptionally Regulates the Expression of CTEN That Is Associated with Prostate Cell Adhesion
Source: PLoS One. 2016 Jan 19;11(1):e0147542. doi: 10.1371/journal.pone.0147542 (PMC4718700; doi:10.1371/journal.pone.0147542)
Supplement: S1 Table — (DOC) [file pone.0147542.s005.doc]

**Table S1. Primers for qPCR and ChIP-qPCR.**

| Primer name | Sequence (5’ to 3’) |
| --- | --- |
| For qPCR | |
| TAp63-forward | AATTTTGAAACTTCACGGTGTGC |
| TAp63-reverse | TCTTTCCAAGAGAAATGAGCTGG |
| ΔNp63-forward | ATTCATATTGTAAGGGTCTCGGG |
| ΔNp63-reverse | GGGCATTGTTTTCCAGGTACAA |
| p63α-forward | TGCAGCATTGTCAGTTTCTTAGC |
| p63α-reverse | TGCTCAATCTGATAGATGGTGGT |
| p63β-forward | TTGCAGCATTGTCAGGATCTGG |
| p63β-reverse | AGAAGGGGAGGAGAATTCGT |
| p63γ-forward | GGAACTCATGCAGTACCTTCCT |
| p63γ-reverse | TTCCTGAAGCAGGCTGAAAGG |
| CTEN-forward | TTTACCACATGCCCAGAGGG |
| CTEN-reverse | CTCTCGGGTGATGTTTGGCT |
| 18S rRNA-forward | CTTAGAGGGACAAGTGGCG |
| 18S rRNA-reverse | ACGCTGAGCCAGTCAGTGTA |
| For ChIP-qPCR | |
| ChIP-CTEN-81-F | CGGAGGGGAGGAGAGAAG |
| ChIP-CTEN+25-R | CTGGGACCAAGACGACTCAG |
| ChIP-CTEN-2420-F | ctcctgacctcgtgatccac |
| ChIP-CTEN-2300-R | TCTCTCACTAAGGGAGAGGG |
| ChIP-CTEN-36621-F | TGCTGCTATTCCACGTCACGTTG |
| ChIP-CTEN-36479-R | CACCTTTAAAGGGAAGTATCAGGCC |
| ChIP-CTEN-8857-F | AGCATGTCCTCTTACTCAGCTGC |
| ChIP-CTEN-8707-R | AAGCTGCTGGACCGTCTGTCCA |
| ChIP-CTEN+7604-F | CTGTTTCTTCAGGCCAGTCAGTC |
| ChIP-CTEN+7755-R | ACTGGTTTTGCCCCAGCTGGC |
| ChIP-CTEN+11036-F | AGGCTGGGGCTCCACAGTCATAA |
| ChIP-CTEN+11176-R | CTGAAGAGTGTGAGTCTTGCCTC |
| ChIP-p21-F | GCAGTGGGGCTTAGAGTGGGG |
| ChIP-p21-R | CAGGCTTGGAGCAGCTACAATTAC |
