# Supplementary material for: ΔNp63α Transcriptionally Regulates the Expression of CTEN That Is Associated with Prostate Cell Adhesion
Source: PLoS One. 2016 Jan 19;11(1):e0147542. doi: 10.1371/journal.pone.0147542 (PMC4718700; doi:10.1371/journal.pone.0147542)
Supplement: S2 Table — (DOC) [file pone.0147542.s006.doc]

**Table S2. p63 ChIP-seq peaks associated with the *CTEN* locus identified by previous studies.**

| **Cell Type** | **Location of peaks*** | | | **Distance to TSS** | | **Site** | **Reference** |
| --- | --- | --- | --- | --- | --- | --- | --- |
|  | **Start** | **End** | **Length** | **From** | **To** |  |  |
| EP156T prostate cell line | 38694054 | 38694746 | 693 | -36813 | -36121 | Upstream | [58] |
| Human primary keratinocyte | 38646661 | 38647031 | 371 | 10903 | 11273 | Intron 2 | [59] |
|  | 38647881 | 38648193 | 313 | 9741 | 10053 | Intron 2 |  |
|  | 38650008 | 38650406 | 399 | 7528 | 7926 | Intron 2 |  |
|  | 38666464 | 38666809 | 346 | -8876 | -8531 | Upstream |  |
| Human primary foreskin keratinocyte | 38646261 | 38647346 | 1086 | 10588 | 11673 | Intron 2 | [60] |
|  | 38647532 | 38648473 | 942 | 9461 | 10402 | Intron 2 |  |
|  | 38647544 | 38648534 | 991 | 9400 | 10390 | Intron 2 |  |
|  | 38649607 | 38650799 | 1193 | 7135 | 8327 | Intron 2 |  |
|  | 38649710 | 38650718 | 1009 | 7216 | 8224 | Intron 2 |  |
|  | 38666145 | 38667189 | 1045 | -9256 | -8212 | Upstream |  |
|  | 38666183 | 38667175 | 993 | -9242 | -8250 | Upstream |  |
| Human primary foreskin keratinocyte | 38646681 | 38647001 | 321 | 10933 | 11253 | Intron 2 | [61] |
|  | 38650119 | 38650372 | 254 | 7562 | 7815 | Intron 2 |  |
|  | 38666433 | 38666840 | 408 | -8907 | -8501 | Upstream |  |
|  | 38694175 | 38694615 | 441 | -36682 | -36242 | Upstream |  |
| HaCaT keratinocyte cell line | 38646380 | 38647132 | 753 | 10802 | 11554 | Intron 2 | [62] |
|  | 38647720 | 38648253 | 534 | 9681 | 10214 | Intron 2 |  |
|  | 38649943 | 38650386 | 444 | 7548 | 7991 | Intron 2 |  |
|  | 38694327 | 38694584 | 258 | -36651 | -36394 | Upstream |  |
|  | 38703661 | 38704192 | 532 | -46259 | -45728 | Upstream |  |

**CTEN* gene is located in chromosome 17q21.2 region and the genomic sequence for *CTEN* gene (human genome NCBI build 37) starts from 38,632,080 bp to 38,657,933 bp (from the terminus of the p arm) on the minus strand.
